# Electrophysiological Excitability and Parallel Fiber Synaptic Properties of Zebrin-Positive and -Negative Purkinje Cells in Lobule VIII of the Mouse Cerebellar Slice

**DOI:** 10.3389/fncel.2018.00513

**Published:** 2019-01-08

**Authors:** Viet T. Nguyen-Minh, Khoa Tran-Anh, Yuanjun Luo, Izumi Sugihara

**Affiliations:** ^1^Department of Systems Neurophysiology, Graduate School of Medical and Dental Sciences, Tokyo Medical and Dental University, Tokyo, Japan; ^2^Center for Brain Integration Research, Tokyo Medical and Dental University, Tokyo, Japan

**Keywords:** Purkinje cell, aldolase C, zebrin II, cerebellum, glutamate transporter, firing pattern, parallel fiber

## Abstract

Heterogeneous populations of cerebellar Purkinje cells (PCs) are arranged into separate longitudinal stripes, which have different topographic afferent and efferent axonal connections presumably involved in different functions, and also show different electrophysiological properties in firing pattern and synaptic plasticity. However, whether the differences in molecular expression that define heterogeneous PC populations affect their electrophysiological properties has not been much clarified. Since the expression pattern of many of such molecules, including glutamate transporter EAAT4, replicates that of aldolase C or zebrin II, we recorded from PCs of different “zebrin types” (zebrin-positive = aldolase C-positive = Z+; and Z−) in identified neighboring stripes in vermal lobule VIII, in which Z+ and Z− stripes occupy similar widths, in the Aldoc-Venus mouse cerebellar slice preparation. Regarding basic cellular electrophysiological properties, no significant differences were observed in input resistance or in occurrence probability of types of firing patterns between Z+ and Z− PCs. However, the firing frequency of the tonic firing type was higher in Z− PCs than in Z+ PCs. In the case of parallel fiber (PF)-PC synaptic transmission, no significant differences were observed between Z+ and Z− PCs in interval dependency of paired pulse facilitation or in time course of synaptic current measured without or with the blocker of glutamate receptor desensitization. These results indicate that different expression levels of the molecules that are associated with the zebrin type may affect the intrinsic firing property of PCs but not directly affect the basic electrophysiological properties of PF-PC synaptic transmission significantly in lobule VIII. The results suggest that the zebrin types of PCs in lobule VIII is linked with some intrinsic electrophysiological neuronal characteristics which affect the firing frequency of PCs. However, the results also suggest that the molecular expression differences linked with zebrin types of PCs does not much affect basic electrophysiological properties of PF-PC synaptic transmission in a physiological condition in lobule VIII.

## Introduction

Purkinje cells (PCs), the sole output neuron of the cerebellar cortex, play an essential role in cerebellar motor and non-motor function through the integration of climbing fiber and parallel fiber (PF) excitatory inputs and inhibitory inputs from molecular layer inhibitory neurons (Ito, 2012). Although the local neuronal circuitry is uniform throughout the cerebellar cortex, PCs are organized into heterogeneous populations based on the expression profile of many molecules, which are distributed in longitudinally-striped patterns in the cerebellar cortex. The striped distribution pattern of heterogeneous populations of PCs is best clarified in the case of zebrin II or glycolytic enzyme aldolase C (Brochu et al., 1990; Fujita et al., 2014). Some 20 aldolase C-positive (Z+) and -negative (Z−) stripes (zebrin stripes) are topographically connected with different areas of the inferior olive and cerebellar nuclei (Voogd et al., 2003; Sugihara and Shinoda, 2004, 2007), and form parallel modules in the olivo-cortico-nuclear loop (Ruigrok, 2011). Expression of several molecules, including EAAT4, a neuronal glutamate transporter (Dehnes et al., 1998), exactly matches with that of aldolase C (Dehnes et al., 1998; Hawkes, 2014), high and low in Z+ and Z− PCs (“zebrin type”), respectively. Expression of other molecules is complementary or partially related to the zebrin type. Although the functional significance of the different expression profiles of these molecules are barely understood, it may be possible that differences in expression of these molecules affect the physiological properties of individual PCs.

Indeed, significant differences in physiological properties have also been reported among PC populations. Z− PCs have significantly higher firing frequencies of simple spikes (Xiao et al., 2014; Zhou et al., 2014), and show a more sustained increase in simple spike activity up to the time of the complex spike (Tang et al., 2017), relative to Z+ PCs in various areas of the cerebellar cortex in *in vivo* studies. In *in vitro* studies, PCs in lobules III-V have been shown to exhibit significantly higher input resistance as well as different variations of firing patterns relative to that of PCs in lobule X (Kim et al., 2012; Cerminara et al., 2015). Furthermore, PCs in lobule III show long term depression more robustly than PCs in lobule X (Wadiche and Jahr, 2005). Notwithstanding, the mechanisms underlying these differences have not been much clarified except for the last case, in which the EAAT4 expression rich in Z+ PCs, which is abundant in lobule X and sparse in lobule III, has been implicated in the rapid lowering of glutamate released by climbing fibers (Wadiche and Jahr, 2005). In the above *in vivo* studies, it is not clear whether these differences are brought about by the molecular expression profile of Z+ and Z− PCs or by specific afferent and local inputs to these PCs. In the seemingly sole *in vitro* study with direct identification of the zebrin type of recorded PCs in EAAT4-eGFP mice (Tsai et al., 2012), the authors reported no significant differences in PF-PC synaptic transmission between Z+ and Z− PCs in normal artificial cerebrospinal fluid (ACSF).

In the present study, we used Aldoc-Venus heterozygous mice (Fujita et al., 2014), in which Aldoc expression is visualized by fluorescent protein expression with a presumably clearer contrast between Z+ and Z− PCs than in EAAT4-eGFP mice, without any other obvious morphological or functional phenotypes. Since the zebrin striped pattern has been clarified in detail (Fujita et al., 2014; Sarpong et al., 2018), it was possible to identify zebrin stripes in longitudinal and transverse slice preparations, in which effects of cerebellar afferent activity are ignorable whereas PF innervation to neighboring Z+ and Z− stripes (Gao et al., 2006) is intact. We focused on lobule VIII, where Z+ and Z− PC populations are arranged into clearly-delineated similarly wide stripes (Fujita et al., 2014). By performing whole-cell patch clamp recording from Z+ and Z− PCs in neighboring stripes in lobule VIII, we made an experimental condition purposely suited to extract differences that are purely related to the zebrin type of PCs. Besides comparing basic electrophysiological properties of Z+ and Z− PCs, we also examined the differences in their PF-PC synaptic transmission, since the EAAT4 expression in PF-PC synapse is linked to the zebrin type and has been implicated in decay of the synaptic current through glutamate uptake (Yamada et al., 1997; Dehnes et al., 1998).

## Materials and Methods

### Ethics Statements

Experimental protocols were approved by the Animal Care and Use Committee (A2017-060C4, A2018-148A) and Gene Recombination Experiment Safety Committee (2012-064C4, 2017-040A) of Tokyo Medical and Dental University.

### Animals

Aldoc-Venus knock-in mouse line of C57BL/6N background (Fujita et al., 2014) was maintained by mating homozygotes. Heterozygotes were produced by mating Aldoc-Venus homozygous males with C57BL/6N females. Postnatal day (P) 18–32 male and female heterozygotes were used in experiments.

### Slice Preparation

Animals were anesthetized with an intraperitoneal injection of an overdose of pentobarbital (0.1 mg/g, Abbott lab, Chicago, IL, USA) and xylazine (0.005 mg/g), and euthanized by cervical dislocation. The cerebellar block was dissected from the extracted brain under ice-cold cutting solution containing (in mM): 125 choline chloride, 3 KCl, 0.1 CaCl_2_, 5 MgCl_2_, 1.25 NaH_2_PO_4_.2H_2_O, 10 D-glucose, 0.4 L-ascorbic acid, 25 NaHCO_3_, 10 HEPES, pH 7.3 and saturated with 95% O_2_ and 5% CO_2_. 200–300 μm transversal and parasagittal slices were cut using a vibratome (PRO7, Dosaka, Osaka, Japan). Slices were initially allowed to recover in cutting solution at 34°C for 5–10 min, after which they were transferred into ACSF containing (in mM): 124 NaCl, 2.5 KCl, 2 CaCl_2_, 1 MgCl_2_, 1.2 NaH_2_PO_4_, 24 NaHCO_3_, and 12.5 glucose and saturated with 95% O_2_ and 5% CO_2_ at 34°C for 30 min, and then allowed to recover in ACSF at room temperature for at least 1 h.

### Identification of Zebrin Stripes in Slice

**Figure 1:**
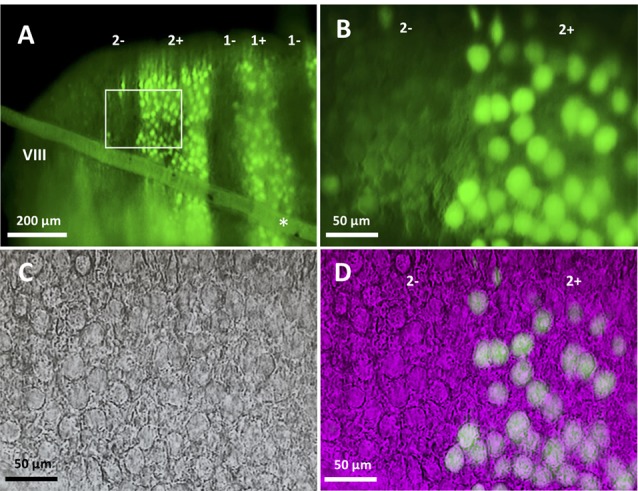
Slice preparation from lobule VIII of the aldolase C-Venus mouse. **(A)** Epifluorescence image of a transverse slice with the 10× objective. The slanted object (asterisk) shows the thread used to hold the slice. Stripe names are indicated in the top. **(B–D)** An enlargement of the region outlined by the white square in **(A)**. Epifluorescence image with the 40× water-immersion objective **(B)**, near-infrared Nomarski interference contrast image **(C)** and superimposition of the epifluorescence and Nomarski images **(D)** showing identification of the zebrin type of individual Purkinje cells (PCs).

Slices were placed in the bottom of the recording chamber, soaked in ACSF, and mounted on the stage of a microscope (BX51IW, Olympus, Tokyo, Japan). In the case of a transversal section, the first section cut from lobule VIII was placed with the apical surface facing the bottom such that the entire PC dendritic arbor was intact in the central part of the slice. Slices were then examined with a 10× objective and epifluorescence optics with a filter for appropriate wavelength selection (Figure 1A). In slices cut in the transversal orientation, it was straightforward in identifying zebrin stripes by referring to the reported aldolase C (zebrin) expression pattern in the Aldoc-Venus mouse (Fujita et al., 2014; see Figures 1, 3). In slices cut in the longitudinal orientation, the distance of the sliced plane from the midline, and location and tilt of Z+ stripes were carefully checked. Z+ stripes were clearly visible even in sagittal slices since Aldoc-Venus mice show a strong contrast of fluorescence expression between Z+ and Z− PCs. Stripes were then identified with reference to the aldolase C stripe pattern which has been mapped upon the unfolded scheme of the cerebellum (Sarpong et al., 2018). For example, stripe 1+ ran nearly in parallel with the plane of the slice throughout lobule VIII in the slice at the midsagittal section. Stripe 2+ ran more laterally in the rostral part and more medially in the caudal part of lobule VIII in the neighboring slice. Stripe 3+ ran medially in the apex of lobule VIII and laterally in the rostral and caudal part of lobule VIII in the same or next laterally neighboring slice. Stripe 4+ was thin and located more laterally in the rostral part, while it was wider and located more medially in the caudal part of lobule VIII, in the same or next laterally neighboring slice. With knowledge of these patterns, we could select a PC from an identified stripe. In a transversal slice, a PC located under a thin layer of granule cells was selected. The optics of the microscope were changed from epifluorescence to near-infrared Nomarski interference contrast system to approach the PC with the electrode.

### Electrophysiological Setups

Slices were constantly superfused with ACSF at room temperature (24°C). PCs were visualized for recording using a 40× water-immersion objective (Figures 1B–D). Patch pipettes had a resistance of 2–5 MΩ when filled with the internal solution consisting of the following (in mM): 120 K-gluconate, 6 NaCl, 10 HEPES, 12 Na_2_-phosphocreatine, 5 EGTA, 1 CaCl_2_, 2 MgCl_2_, 2 MgATP, 0.5 NaGTP, pH 7.3, adjusted with KOH; osmolality ~290 mosmol/kgH_2_O. Signals from the patch pipette were recorded with a MultiClamp 700B amplifier (Molecular Devices, San Jose, CA, USA), digitized at 10–20 kHz and filtered at 2–5 kHz with a Digidata 1440A analog-to-digital converter (Molecular Devices). Whole-cell recording was made at a holding potential of −70 to 75 mV in current clamp and voltage clamp modes by injecting holding current <500 pA. Access resistance, which was monitored to check stability throughout the recording, was <10 MΩ. Experiments in which holding current was >500 pA or access resistance changed by >15% were not included for analysis. Recorded data were analyzed in Clampfit 10.7 (Molecular Devices).

### Identification of the Spike Response Type

Sagittal and transverse slice preparations from P20 to P30 mice were used. Recording was done under current clamp mode with the membrane potential set to −70 to 75 mV by injecting holding current <500 pA in current clamp mode. ACSF containing 100 μm picrotoxin (C0375, Tokyo Chemical Industry Co., Tokyo, Japan) and 2 mM kynurenic acid (H0303, Tokyo Chemical Industry Co., Tokyo, Japan) was superfused to block spontaneous synaptic activities. Firing patterns were determined in response to depolarizing current pulses (100–1,000 pA) of 1,000 ms injected from hyperpolarized holding potentials.

### Measurement of Input Resistance

Sagittal and transverse slice preparations from P20 to P30 mice were used. Recording was done under current clamp mode with the membrane potential set to −70–75 mV by injecting holding current <500 pA in current clamp mode. ACSF containing 100 μm picrotoxin and 2 mM kynurenic acid was superfused. Input resistance was obtained from the membrane potential response to the negative current injections of four different intensities (−600, −500, −400 and −300 pA) in current clamp mode.

### Firing Property Analysis

Sagittal slice preparations were made from P20 to P30 mice. Recordings were made from Z+ and Z− PCs in stripes between 1+ and 3− in lobule VIII under current clamp mode with the membrane potential set to approximately −70 mV. ACSF containing 100 μm picrotoxin and 2 mM kynurenic acid was superfused. After identifying its response pattern as the tonic type, 20 current steps of 1,000 ms duration (20 pA incremental steps from 0 pA to 380 pA). The number of action potentials during the 1,000 ms period was obtained for each current injection step.

### Recording PF-PC Synaptic Current

Sagittal slice preparations from P18 to P25 and P28–32 mice were used. Recording was done under voltage clamp mode at a holding potential of −70 mV. ACSF containing 100 μm picrotoxin was superfused to block activities of inhibitory synapses in some experiments. ACSF containing 100 μmol cyclothiazide (CTZ, ab120061, Abcam, Cambridge, UK) and 100 μm picrotoxin was superfused to block desensitization of α-amino-3-hydroxy-5-methyl-4-isoxazolepropionic acid receptor (AMPAR) additionally in other experiments. Stimulation electrode (glass pipette with the tip diameter of 5–10 μm, filled with ACSF) was placed in the deep portion of the molecular layer (~25–50 μm to the PC layer, Roth and Häusser, 2001) to obtain consistent fast decay time course of the synaptic current (half decay time of about 8 ms, see Figure 4A). For stimulation, negative pulses (duration, ~0.1 ms) were given between stainless wires in the glass pipette and in the bath. Stimulus intensities were adjusted to observe an excitatory postsynaptic current (EPSC) amplitude of 100–150 pA from the whole-cell patch pipette. PF stimulation was confirmed by slow rising of EPSC, variable EPSC amplitude nearly linearly related to the stimulus intensity and paired-pulse facilitation (see Figure 4B).

**Figure 2:**
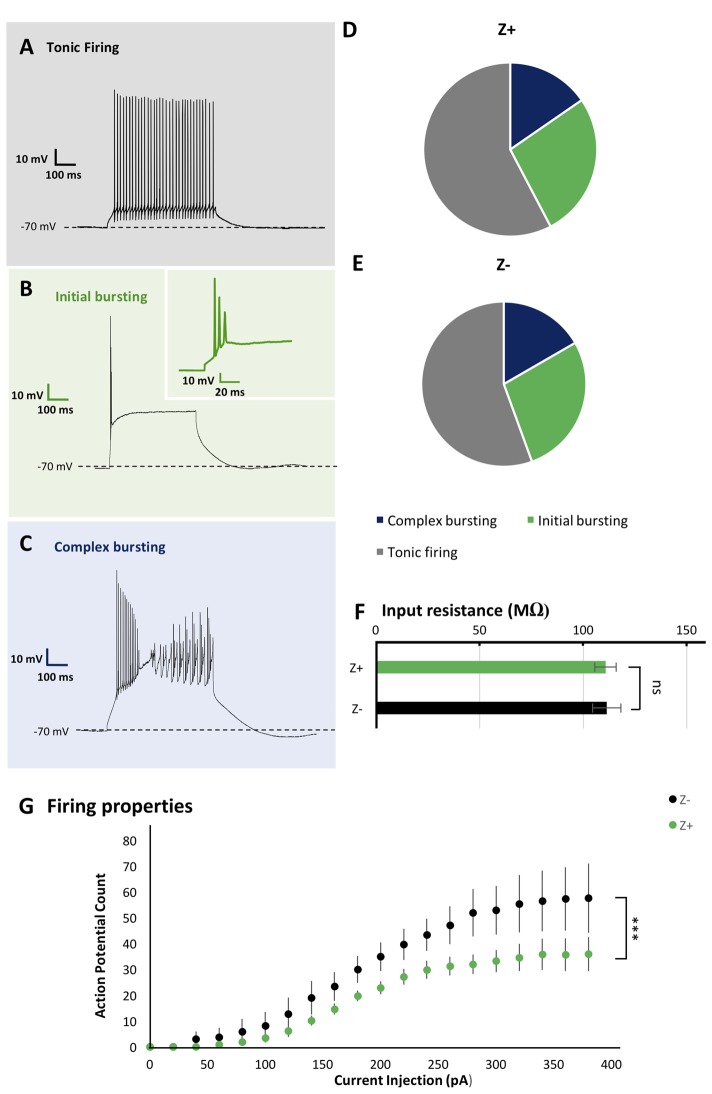
Similar firing pattern variation and input resistance between Z+ and Z− PCs in lobule VIII. **(A–C)** Types of firing patterns in response to depolarizing current injection in three PCs. They represent tonic firing type **(A)**, initial bursting type **(B)** and complex bursting type **(C)**, respectively.** (D,E)** Pie charts show the relative proportion of each type of firing patterns Z+ (*n* = 24) and Z− (*n* = 19) PCs, respectively. **(F)** Comparison of input resistance between Z+ (*n* = 19) and Z− (*n* = 14) PCs. **(G)** Plot of the mean action potential count during the 1,000 ms current injection step against the current injection intensity, obtained for 10 Z+ (green) and 10 Z− (black) PCs of the tonic firing type (ns, not significant; ***p <0.001).

**Figure 3:**
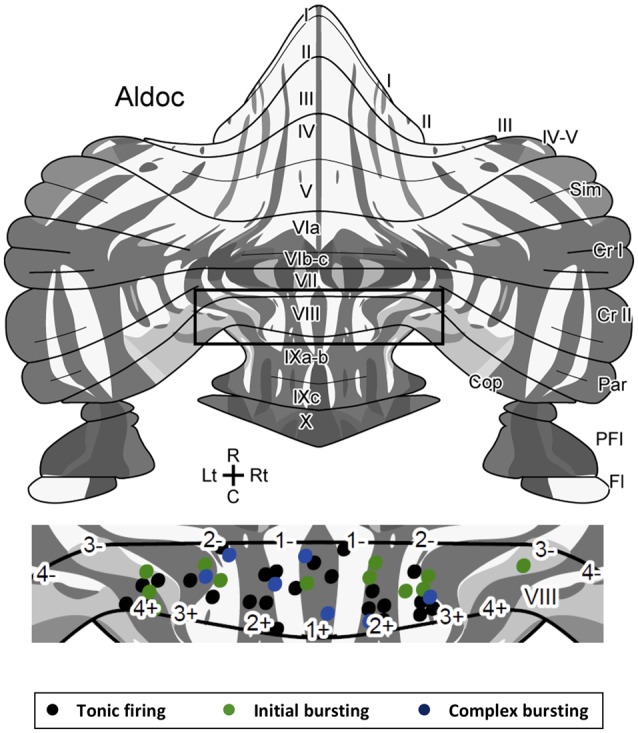
Location of Z+ and Z− PCs with different types of firing patterns mapped on the unfolded scheme of the mouse cerebellar cortex with the zebrin stripe. Forty-three PCs are mapped in the lobule VIII area (center) of the scheme of the zebrin stripe of the mouse cerebellum (top, Sarpong et al., 2018) with a color coding of firing pattern types (bottom). Square in the top scheme indicates the lobule VIII area that is magnified in the center.Abbreviations, I–X, lobules I–X; a–c, sublobules a–c; Cop, copula pyramidis; Cr I, crus I; Cr II, crus II; Fl, flocculus; Par, paramedian lobule; PFl, paraflocculus; Sim, simple lobule.

**Figure 4:**
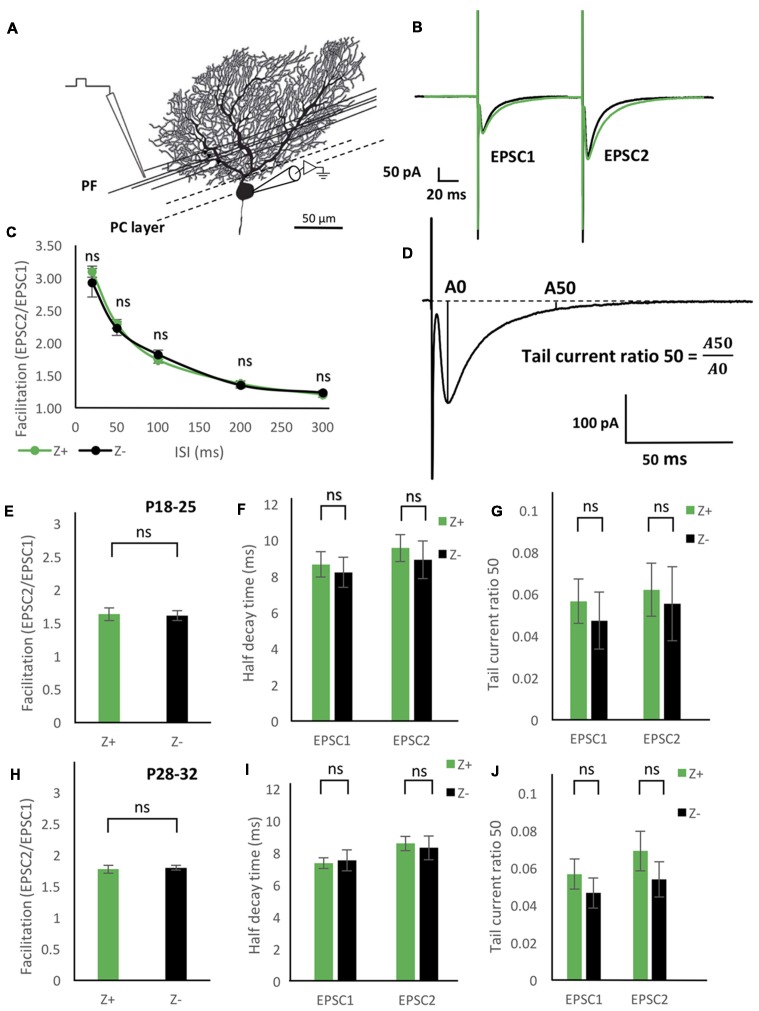
Similar excitatory postsynaptic current (EPSC) properties of the parallel fiber (PF)-PC synapse between Z+ and Z− PCs. **(A)** Illustration of the experiment. The stimulation electrode was placed in the deep molecular layer 25–50 μm from the PC layer. **(B)** Average of PF-PC EPSCs recorded in Z+ (green, *n* = 8) and Z− (black, *n* = 8) PCs. **(C)** Time course of the facilitation, which was defined as the amplitude of the EPSC evoked by the second stimulus (EPSC2) divided by the amplitude of the EPSC evoked by the initial stimulus (EPSC1; *n* = 7 Z+ and 7 Z−). **(D)** Schematic showing definition of “tail current ratio 50,” which is the ratio of the EPSP amplitude at 50 ms after the peak (A50) vs. the EPSC peak amplitude (A0). **(E–G)** Facilitation with the paired pulse interval of 100 ms **(E)**, half-decay time **(F)**, and tail current ratio 50 **(G)** of PF-PC EPSC in P18–25 mice (*n* = 8 Z+ and 8 Z−), respectively. In **(F,G)**, values for the EPSC1 and EPSC2 are shown. **(H–J)** Same measurements as **(E–G)** performed in P28–32 mice (*n* = 8 Z+ and 8 Z−; ns, not significant).

### Statistical Analysis

Measured values are shown with the mean ± standard error of the mean (SEM). The average and SEM were plotted in figures. Firing properties were analyzed using two-way ANOVA, while other statistical analyses were performed using one-way ANOVA. Differences were considered significant at *p* < 0.05. Single, double and triple asterisks indicate *p* < 0.05, *p* < 0.01, *p* < 0.001.

## Results

### Firing Pattern of PC Was Not Correlated to the Zebrin Type and Location in Lobule VIII

In slice preparation obtained from Aldoc-Venus mice at the second and third postnatal week, fluorescence of individual PCs was clearly visible with 10× and 40× objectives, and consequently, it was straightforward to identify the longitudinally-arranged pattern of Z+ and Z− stripes of PC distribution (Figure 1). Whole-cell patch clamp recording was performed from single Z+ or Z− PCs within an identified stripe in lobule VIII.

As the basic electrophysiological property that may be correlated with regional difference or striped pattern, the type of spike response to step-wise current injection and input resistance were examined. These properties are reported to be significantly different between PCs in lobules III-V and lobule X (Kim et al., 2012) and thus suggested to be possibly correlated with the zebrin type (Cerminara et al., 2015). We investigated PC firing patterns in vermal area of lobule VIII. It was revealed that PCs generated a particular type of spike response, with suprathreshold current injection, which could be classified into multiple types as described previously (Kim et al., 2012; Cerminara et al., 2015). Among 44 PCs recorded in lobule VIII, tonic firing (Figure 2A), initial bursting (Figure 2B) and complex bursting (Figure 2C) types were found in 57% (*n* = 25), 16% (*n* = 7) and 27% (*n* = 12) PCs, respectively. However, no gap firing pattern, which has been observed in lobule X PCs (Kim et al., 2012), was observed.

The sample of 44 PCs included 26 Z+ and 18 Z− PCs. The tonic firing, initial bursting and complex bursting types were observed in 58% (*n* = 15), 27% (*n* = 7) and 15% (*n* = 4) of Z+ PCs (Figure 2D), and in 55% (*n* = 10), 28% (*n* = 5) and 17% (*n* = 3) of Z− PCs (Figure 2E). The results showed no clear differences in the occurrence of different types of spike response between Z+ and Z− PCs in lobule VIII. This was an indication that the zebrin type of PCs was not much correlated with the firing pattern of PCs in lobule VIII.

Since there are multiple Z+ and Z− stripes in lobule VIII, we mapped the location of the recorded PCs on the scheme of aldolase C stripes mapped on the unfolded mouse cerebellar cortex (Sugihara and Quy, 2007; Fujita et al., 2014; Sarpong et al., 2018) to further examine the distribution of PCs with respect to the different firing patterns. PCs with all of the three firing types were randomly distributed in various stripes as shown (Figure 3). We did not observe any particular relationship between the distribution of firing types and the location of Z+/Z− stripes or in the anterior-posterior/medial-lateral axes.

### Input Resistance of PCs Was Not Correlated With the Zebrin Type

Input resistance relates to neuronal size, shape and resting membrane conductance. Previous studies have shown that PCs in lobules III-V have significantly larger input resistance that those in lobule X (Kim et al., 2012). However, it is not clear whether this difference is related to Z types of PCs, which are differently distributed in these lobules. Therefore, we measured the input resistance in 19 Z+ and 14 Z− PCs in lobule VIII. The obtained values, 110.8 ± 5.1 MΩ for Z+ PCs and 111.3 ± 6.8 MΩ for Z− PCs were not significantly different between the two populations (*F*_(1,31)_ = 0.05, *p* = 0.95, one-way ANOVA). The result indicates that the input resistance of PCs were not correlated with the zebrin type in lobule VIII (Figure 2F).

### Comparison of Action Potential Count in Tonic Firing Type PCs

*In vivo* studies have reported higher firing frequency in Z− PCs than in Z+ PCs (Xiao et al., 2014; Zhou et al., 2014). Therefore, we examined the intrinsic firing properties of Z+ and Z− tonic firing type PCs in lobule VIII (10 Z+ and 10 Z− PCs in stripes 1+ to 3−), which comprised the largest population among the three types of firing patterns of Z+ and Z− PCs in lobule VIII (above), in a separate set of experiments with P22 and P23 mice. Although no differences were found in the rheobase, the lowest current intensity needed to produce firing between these Z+ and Z− PCs examined (data not shown), current injection steps produced a higher count of action potential firing in Z− PCs than in Z+ PCs in the input-output relationship (two-way repeated-measures ANOVA starting at 0 pA injection, factor zebrin type, *F*_(1,18)_ = 37.41; *p* < 0.001; Figure 2G).

### Comparison of PF-PC EPSC Properties Between Z+ and Z− PCs

PFs run in the transverse plane along the major axis of a folium and excite PC dendrites along an extension of about 3 mm to cross multiple zebrin stripes and make excitatory synaptic connection to both Z+ and Z− PCs (Arata and Ito, 2004; Gao et al., 2006; Ito, 2006; Hoxha et al., 2016). Thus, PCs in neighboring zebrin stripes receive innervation from a similar population of PFs. Therefore, if there exist any differences in the property of PF-PC synapses between nearby Z+ and Z− PCs in the same lobule, they would indicate differences related to the zebrin type.

To study functional difference in PF-PC synaptic transmission between Z+ and Z− PCs, we first examined the degree of facilitation of EPSC of PF-PC synapse (Figures 4A,B), one of the essential plastic characteristics of synaptic transmission. The ratio of the amplitudes of the first and second EPSC evoked by paired pulse stimulation with an interval of 20 ms, 50 ms, 100 ms, 200 ms and 300 ms was measured in mice at P18–25. Results from 7 Z+ and 7 Z− PCs showed no significant differences at any paired pulse interval (Figure 4C). This indicated that PF-PC synapse exhibit similar degree and time course of facilitation between Z+ and Z− PCs (Figure 4C), suggesting that presynaptic mechanisms of PF-PC synapse were not much affected by the zebrin type of postsynaptic PCs.

Among the molecules that have expression pattern linked to the zebrin type, EAAT4, a glutamate transporter, is expressed in PC dendritic spines, which form synaptic contact with PFs and are surrounded by astrocytes (Yamada et al., 1997; Dehnes et al., 1998; Lehre and Danbolt, 1998; Danbolt, 2001), and involved in the removal of low concentrations of glutamate (Takayasu et al., 2005). Therefore, we focused on the decay process of the EPSC of PF-PC synapse between Z+ and Z− PCs. We prepared two groups of different ages (P18–25 and P28–32) in another set of experiments of paired pulse stimulation of PFs (*n* = 8 Z+ and 8 Z− PCs at P18–25, *n* = 8 Z+ and 8 Z− PCs at P28–32), since EAAT expression level is dependent on postnatal date; effects of EAAT4 may be better observed at P28–32, when expression reaches the adult level, compared to P18–25, when it is still lower (Furuta et al., 1997). In this set of experiments, we did not observe significant differences in facilitation, i.e., the ratio of the amplitudes of EPSCs evoked by paired pulses with an interval of 100 ms, between Z+ and Z− PCs in the two age groups (*F*_(1,14)_ = 0.03, *p* = 0.86; *F*_(1,14)_ = 0.1, *p* = 0.76, one-way ANOVA respectively, Figures 4E,H). No significant difference was observed in “half decay time,” time for the change of the amplitude from the peak to the half of the peak value, of the initial and second EPSC, between Z+ and Z− PCs in the two age groups (*F*_(1,14)_ = 0.16, *p* = 0.7; *F*_(1,14)_ = 0.06, *p* = 0.8, one-way ANOVA, respectively, Figures 4F,I). Finally, no significant difference was observed in “tail current ratio,” the amplitude of EPSC 50 ms after the peak (Figure 4D; Takayasu et al., 2005), of the initial and second EPSC, between Z+ and Z− PCs in the two age groups (*F*_(1,14)_ = 0.29, *p* = 0.60; *F*_(1,14)_ = 0.77, *p* = 0.39; one-way ANOVA, respectively, Figures 4G,J). The results revealed that the different expression levels of EAAT4 between Z+ and Z− PCs do not affect the time course of PF-PC EPSC decay. This was against our original hypothesis that Z+ PCs might show faster EPSC decay because of higher expression of EAAT4, which uptakes low concentration glutamate (Takayasu et al., 2005).

### Blocker of AMPAR Desensitization Did Not Distinguish PF-PC EPSCs Between Z+ and Z− PCs

**Figure 5:**
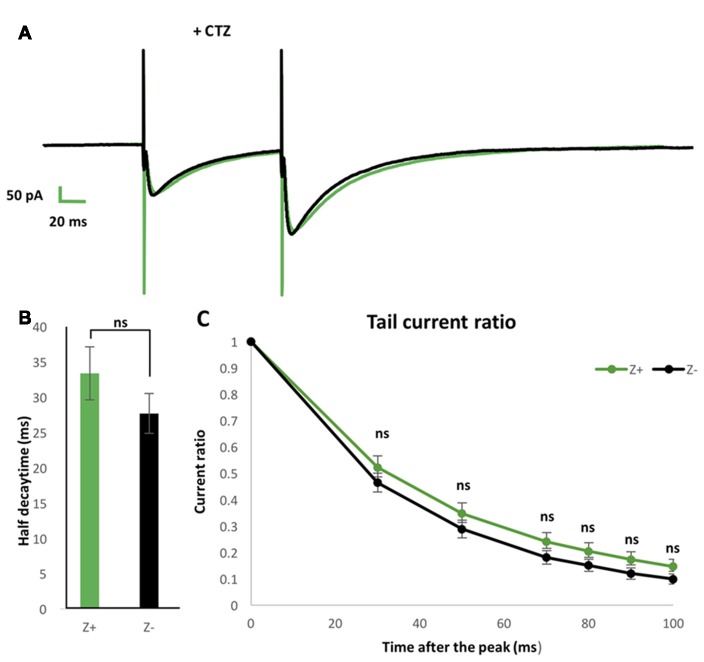
Similar EPSCs decay kinetics of PF-PC synapse between Z+ and Z− PCs in the presence of blocker of α-amino-3-hydroxy-5-methyl-4-isoxazolepropionic acid receptor (AMPAR) desensitization 100 μmol cyclothiazide (CTZ). **(A)** Average of PF-PC EPSCs recorded in Z+ (green, *n* = 9) and Z− (black, *n* = 9) PCs in the presence of 100 μmol CTZ. **(B)** Half decay time of the EPSC evoked by the second stimulus. **(C)** Plots of tail current ratios, ratio of the EPSC amplitude at 30, 50, 70, 80, 90, 100 ms after the peak vs. the peak EPSC amplitude, of the EPSC evoked by the second stimulus. Interval of the paired stimuli was set at 100 ms (ns, not significant).

To further examine possible EAAT4-dependent differences in PF-PC synaptic transmission between Z+ and Z− PCs, we added 100 μmol CTZ in the bath to reduce desensitization of AMPAR (Partin et al., 1994) in another set of experiments of paired pulse stimulation of PFs with slice cut from P28 to P32 mice (*n* = 9 Z+ and 9 Z− PCs). This condition was supposed to enable a sensitive detection of glutamate removal process in the PF-PC synapse, as observed with marked increase of EPSC tail current in EAAT4-deficient mice (Takayasu et al., 2005). Indeed, time course of the EPSC decay was significantly longer under CTZ application (half decay time about 30 ms) than in normal ACSF (about 9 ms, Figures 4F,I) both in Z+ and Z− PCs. The time course of decay of the second EPSC appeared slightly slower in Z+ PCs than Z− PCs (Figure 5A). However, statistical analysis showed no significant difference between them when the amplitude was compared at given times after the peak (*F*_(1,16)_ = 1.09, *p* = 0.31; *F*_(1,16)_ = 1.24, *p* = 0.28; *F*_(1,16)_ = 1.99, *p* = 0.18; *F*_(1,16)_ = 1.83, *p* = 0.19; *F*_(1,16)_ = 2.16, *p* = 0.16; *F*_(1,16)_ = 2.01, *p* = 0.17, one-way ANOVA, for 30, 50, 70, 80, 90, 100 ms after EPSC peak, respectively; Figure 5C). Half decay time measured in the second EPSC did not show significant difference between Z+ and Z− PCs, either (*F*_(1,16)_ = 1.47, *p* = 0.24, one-way ANOVA, Figure 5B). The results further indicated that higher EAAT4 expression did not make EPSC decay faster in the PF-PC synapse in Z+ PCs.

## Discussion

The present study showed that there are no significant differences in input resistance, occurrence probability of types of firing patterns or in kinetics of PF-PC synaptic current between Z+ and Z− PCs in neighboring zebrin stripes in lobule VIII, in Aldoc-Venus mouse cerebellar slices. However, the intrinsic firing frequency of the tonic firing type was significantly higher in Z− negative PCs than Z+ PCs. The implications of the present results and possible functional significance of zebrin types in PCs are discussed.

### Use of Aldoc-Venus Mice in Phenotype Study of Z+ and Z− PCs

Since many molecules are heterogeneously expressed in PC populations, it is an essential question what functional differences PC populations attain because of the heterogeneous molecular expression. However, this question is not simple to test, since the functional property of individual PCs is not solely dependent on their intrinsic molecular expression profiles but also on their cellular morphology, synaptic inputs from local circuits and afferent projections, and the environmental interaction between nearby extracellular and cellular components.

To address this question simply, we focused on a particular molecule, aldolase C or zebrin II, the expression pattern of which probably matches with the expression pattern of the largest number of other molecules. Aldoc-Venus mice facilitate a clear recognition of Z+ and Z− PCs, which are arranged in the longitudinal striped pattern at adult and at stages as early as the second postnatal week (Fujita et al., 2014).

By making slice preparations from Aldoc-Venus mice, we could sample identified Z+ and Z− PCs from identified stripes in an identified lobule. Lobule VIII, on which we focused in this study, contained a comparable number of Z+ and Z− PCs (Figure 3), different from lobules III or X. By sampling both Z+ and Z− PCs from neighboring stripes in lobule VIII, we eliminated the effects of environmental factors, if any. By using slice preparations, we excluded the effects of different inputs to Z+ and Z− PCs arising from climbing fibers. Thus, our preparation is better suited to detect electrophysiological properties that are directly related to the zebrin type of PCs than previously reported preparations.

### Zebrin Type of PCs Is Linked to Some Aspects of Electrophysiological Excitability of PCs in Lobule VIII

Many types of heterogeneous electrophysiological properties have been reported among cerebellar PCs (Cerminara et al., 2015). The main focus of this study was to clarify the properties that are tightly linked to the zebrin type of PCs.

Input resistance of PCs in lobules III-V is significantly higher than that of PCs in lobule X (Kim et al., 2012). This difference is even sharper when tonic firing PCs in these lobules are compared (Kim et al., 2012). Since the majority of PCs are Z− in lobules III-V while almost all PCs are Z+ in lobule X, their finding may suggest that Z− PCs have larger input resistance. However, the present results showed that the zebrin type of PCs was not tightly linked with different input resistances in lobule VIII, suggesting that differences in input resistance might reflect lobular organization and other factors. Recently, it has been reported that the morphology of PC dendritic arbor is significantly different among lobules and among locations in a lobule (Nedelescu et al., 2018). Such morphological differences may affect the PC input resistance rather than expression of molecules that is linked to the zebrin type.

PCs show different types of spiking activity in response to step-wise current injections (Kim et al., 2012). Two-thirds and one-third of PCs show tonic firing and complex bursting patterns in lobules III–V, while four groups of about one-fourth of PCs show tonic firing, complex bursting, initial bursting and gap firing patterns, respectively, in lobule X (Kim et al., 2012). Since the majority of PCs are Z− in lobules III-V while almost all PCs Z+ in lobule X, this difference may suggest that the occurrence probability of different spiking activity may be related to the zebrin type (Cerminara et al., 2015). However, the present results showed otherwise in lobule VIII. Although ionic conductance mechanisms that lead to the different spiking activity have not been fully understood, A-type K channels are responsible for the initial bursting response (Kim et al., 2012). However, we found that the firing frequency was higher in tonic firing type Z− PCs than in tonic firing type Z+ PCs. Although the underlying mechanisms for this difference is not clear, we speculate that some differences in expression level of voltage-dependent ion channels may be linked with the zebrin type of PCs. PC firing pattern is also related to intracellular Cl− concentration and expression of glutamate transporter, including EAAT4, which is highly expressed in Z+ PCs, that can affect intracellular Cl− concentration (Rabenstein et al., 2018). Our internal solution (K-gluconate-based solution) contained small fixed amounts of Cl−, which may have covered some effects mediated by Cl− between Z+ and Z− PCs.

### Molecular Expression Differences in Zebrin Stripes Does Not Affect EPSC Kinetics in the PF-PC Synapse in Lobule VIII

In the present study, we made a systematic observation of PF-PC synaptic transmission between Z+ and Z− PCs in lobule VIII. A PF runs a few millimeters in the molecular layer. The experimental fact that the lateral spread of transverse beam activity evoked by PF stimulation to all parasagittal bands under suppression of inhibitory synaptic transmission (Gao et al., 2006) indicates that a PF makes synaptic contact to both Z+ and Z− PCs. Therefore, zebrin type-dependent electrophysiological differences in PF-PC synaptic transmission, if there are any, would be a result of the zebrin type-related property of postsynaptic PCs. On the contrary, zebrin type-dependent electrophysiological differences in climbing fiber-PC synaptic transmission may be a result of either different presynaptic property of climbing fibers (Paukert et al., 2010), since Z+ and Z− PCs are innervated by distinct population of climbing fibers (Voogd et al., 2003; Sugihara and Shinoda, 2004), or zebrin type-related property of postsynaptic PCs. Therefore, we are focused on the PF-PC synaptic transmission in the present study.

Among synaptic molecules, EAAT4 is expressed at a higher level in Z+ PCs than in Z− PCs, with a three-times difference in protein amount detected by gold particle counting in immunostaining under electron microscopy (Dehnes et al., 1998). EAAT4 expression is present in PCs at embryonic day 13 and increases to a maximum adult level at P26 in the mouse (Furuta et al., 1997; Yamada et al., 1997). EAAT4 is present in PCs at and around PF-PC and climbing fiber-PC synapses. It is located most densely at the side of the spine and on the dendritic membrane around the spine facing astrocytes or Bergmann glia, and less densely on the postsynaptic membrane of the spine (Dehnes et al., 1998; Danbolt, 2001). EAAT4 is involved in glutamate uptake from the extracellular cellular space (Takayasu et al., 2005) and glutamate-gated Cl− permeability (Fairman et al., 1995). In synaptic transmission, EAAT4 has been regarded to be responsible for the removal of low levels of glutamate that remains in the extrasynaptic region and hence for the time course of late decay of the EPSC (Takayasu et al., 2005).

In relation to the functional distinction between Z+ and Z− PCs, the properties of EAAT4 described above suggest that Z+ PCs may have faster decay of PC-PF EPSC, due to faster reuptake of glutamate from the synaptic cleft, than Z− PCs. However, kinetics of EPSC decay time course were not significantly different between Z+ and Z− PCs, in normal ACSF at P18–25, P28–32 and in ACSF with CTZ, a blocker of desensitization of AMPAR, at P28–32 in lobule VIII. These findings showed no evidence of faster glutamate removal or faster EPSC decay time course in Z+ PCs than in Z− PCs, even with the increased glutamate release by the paired stimulus and with the enhanced glutamate sensitivity of the glutamate receptor. This result generally agreed with that of Tsai et al. (2012), who have found similar release properties of PF-PC synapse in Z+ and Z− PCs and have concluded that the variation of endogenous EAAT4 expression does not alter the time course of EPSCs, presumably in lobules III and IV, although they have not explicitly identified the lobule. As a sole observed difference between Z+ and Z− PCs, Tsai et al. (2012) reported that the current required to evoke a half-maximum response was significantly greater in Z+ PCs with CTZ. We did not test half-maximum with CTZ response in the present study. As a whole, the results indicate that EAAT4-dependent clearance of glutamate occurs in a way that does not significantly affect the decay time course of EPSC in PF-PC synaptic activity in conjunct with other synaptic activity. Consequently, the results suggest that PF-PC synaptic transmission does not receive a crosstalk from other PF-PC synapses (Lehre and Danbolt, 1998; Takayasu et al., 2005) through spill-over of glutamate in either Z+ or Z− PCs. Presumably, other glutamate transporters GLAST and GLT, which are expressed highly in astrocytes or Bergmann glia processes (Lehre and Danbolt, 1998) play the major role in the removal of glutamate in inconjunct PF-PC synaptic transmission. We speculate that the location of EAAT4 expression, mainly at the side of the dendritic spine facing astrocytes (Dehnes et al., 1998), is functionally too far to affect the glutamate level for AMPAR response in the PF-PC synapse. Our discussion above may be at variance with the results of *in vitro* experiments which showed a significant decrease in late EPSC amplitude of PF-PC synapse in EAAT4 knock-outs (Takayasu et al., 2005). This point may require further investigation concerning the localization of EAAT4, effects of its mutation, and intracellular storage (Dehnes et al., 1998) in a comparison between Z+ and Z− PCs.

Regarding the high affinity for glutamate by EAAT4 (Fairman et al., 1995), other functions may still be expected for the high EAAT4 expression in PF-PC synapse in Z+ PCs: (1) it may isolate the PF-PC synapse from spillover of glutamate released in other surrounding synapses, especially the climbing fiber-PC synapses (Szapiro and Barbour, 2007); (2) it may function as Cl− permeability (Fairman et al., 1995) that shunts inward current when it is exposed to spillover of glutamate; and (3) it may control extrasynaptic signaling to Bergmann glial cells (Tsai et al., 2012). Our recording condition was not tuned to detect Cl− permeability. Hence, further studies would be required to clarify these effects.

### Possible Functional Significance of Zebrin Types in PCs

The present study showed that zebrin type was related with basic excitability of PCs to some extent, but not directly related with PF-PC synaptic transmission kinetics, in lobule VIII. The results suggest that the significantly higher simple spike firing frequency of Z− PCs relative to Z+ PCs, which has been observed in various cerebellar areas in *in vivo* preparation (Xiao et al., 2014; Zhou et al., 2014), may be partly because of the difference in intrinsic properties linked with the zebrin type. However, the other possibility such as different input patterns to Z− and Z+ PCs cannot be excluded. The present study did not cover differences in long term synaptic plasticities or inter-synaptic interactions between Z+ and Z− PCs, which remain to be clarified. Concerning interaction between the PF-CF synapse and the climbing fiber-PC synapse, the efficiency of occurrence of long term depression in the PF-PC synapse is dependent on zebrin types through EAAT4-dependent glutamate uptake (Wadiche and Jahr, 2005). Besides the long term depression, simple spike activity was modulated by occurrence of complex spikes and this modulation is larger in Z+ PCs than in Z− PCs (Tang et al., 2017). The mechanisms involved in this function are yet to be studied.

## Data Availability

The raw data supporting the conclusions of this manuscript will be made available by the authors, without undue reservation, to any qualified researcher.

## Author Contributions

All authors had full access to all the data in the study and take responsibility for the integrity of the data and accuracy of the data analysis. VN-M and IS: study concept and design and critical revision of the manuscript for important intellectual content. VN-M: acquisition of data. VN-M, KT-A and IS: analysis and interpretation of data. VN-M, YL and IS: drafting of the manuscript.

## Conflict of Interest Statement

The authors declare that the research was conducted in the absence of any commercial or financial relationships that could be construed as a potential conflict of interest.
